# Impact of novel coronavirus disease (COVID-19) on Egyptian dentists’ fear and dental practice (a cross-sectional survey)

**DOI:** 10.1038/s41405-020-00047-0

**Published:** 2020-10-12

**Authors:** Mariam Mohsen Aly, Marwa Aly Elchaghaby

**Affiliations:** Pediatric Dentistry and Dental Public Health, Faculty of Dentistry, Cairo University, Giza, Egypt

**Keywords:** Dental epidemiology, Occupational health

## Abstract

**Objectives:**

This study aimed to evaluate the fear of infection among Egyptian dentists practicing during the current coronavirus disease 2019 (COVID-19) pandemic and to explore the dentist’s knowledge about guidelines to fight the virus and to assess various modifications in dental practice.

**Methods:**

An online survey was submitted to dental professionals. Data were collected through a validated questionnaire consisting of 23 closed-ended questions. The gathered data were statistically analyzed.

**Results:**

An overall 216 dentists completed the survey. A total of 200 (92.6%) dental professionals were afraid of becoming infected with COVID-19 while 196 (90.7%) became anxious to treat patients showing suspicious symptoms. The majority of the participants were aware of the mode of transmission of COVID-19 and a lot of them were updated with the current Disease Control and Prevention (CDC) or World Health Organization (WHO) guidelines for cross-infection control.

**Conclusions:**

COVID-19 pandemic has a significant impact on dental professionals.

## Introduction

In December 2019, patients with pneumonia of unknown cause were detected in the city of Wuhan and within a couple of weeks, it reached other parts of the world, which generated a major public health crisis worldwide and imposed a challenge to healthcare systems across the six continents.^1,2^

The World Health Organization (WHO) named the Chinese outbreak COVID-19 and declared it to be a public health emergency of international concern posing a high risk to countries with vulnerable health systems.^3,4^

The spread of coronavirus (COVID-19) has posed significant challenges for dentistry and medicine, and dental and medical schools, in all affected countries.^5^ The reaction speed and response type to this disease have been very variable around the world according to different healthcare systems, economies, and political views.^6^

Due to the nature of dental settings, the risk of cross-infection may be huge between dental practitioners and patients through direct transmission by a sneeze, cough, or droplet inhalation, or contact transmission such as ocular contact or through mucous membranes of the eyes and nose and saliva.^3^

For dental clinics and hospitals in regions and countries that are potentially influenced by COVID-19, firm, and effective infection control protocols are crucially required. This is a result of the unique attributes of dental procedures where an enormous number of droplets and aerosols could be generated, the usual protective measures in daily clinical practice are not effective enough to counter the spread of COVID-19, especially when patients are in the incubation period and are unaware that they are infected or prefer to conceal their infection.^7^

Different practical guidelines were recommended for dental professionals by the Centers for Disease Control and Prevention (CDC), the American Dental Association (ADA), and the WHO to control the spread of COVID-19 and like other contagious infections, these recommendations include personal protective equipment, hand washing, detailed patient evaluation, rubber dam isolation, anti-retraction handpiece, mouth rinsing before dental procedures, and disinfection of the clinic.^8^

In a pandemic, fear raises anxiety and stress levels in healthy persons and escalates the symptoms. The number of persons whose mental health is affected tends to be greater than the number of persons affected by infection.^9^

Fear and anxiety are powerful emotions that may be associated with the overwhelming reports on the pandemic by social, electronic, and print media, the absence of well-structured scientific evidence and knowledge about COVID-19, time-consuming screening and diagnostic methods, insufficient personal protection equipment, unclear treatment, and immunization.^5^

Mild anxiety is natural and fosters preventive and safeguarding behavior. Although the ADA has published preventive guidelines, the majority of dentists are still reluctant and fearful of treating patients in such a situation.^10^

Before effective approaches to reinforce dental professionals can be developed, it is crucial to recognize their specific sources of anxiety and fear. Focusing on addressing those concerns, rather than teaching general approaches to reduce stresses, should be the main focus of support efforts.^11^

This study aimed to evaluate the fear of infection among Egyptian dentists practicing during the current COVID-19 pandemic and to explore the dentist’s knowledge about guidelines to fight the virus and to assess various modifications in dental practice.

## Methods

### Study design and setting

The present study was a cross‐sectional study of a random sample of Egyptian dental professionals. The STROBE guidelines were used to ensure the reporting of this observational study. Data were collected via an online survey link and received a response through an online survey submission.

### Participants

Any Egyptian dentist having a bachelor of dentistry or equivalent degree was invited to participate while dental students were not included in this survey.

### Trial registration

This study has been registered with clinicaltrials.gov under the title: fear and practice modification among dentists during COVID-19 pandemic with an identifier: NCT04383626.

### Study size

To assess the prevalence of fear of becoming infected, the sample size was calculated (http://www. nss. gov. au/ nss/ home.nsf/ pages/ Sample+ size+ calculator) using the following assumptions: confidence level = 95% CI = 5%, the total number of dentists with an estimated percentage of fear of becoming infected = 87% according to Ahmed et al.^10^ The number was increased by 25% to allow for nonresponse. The required sample size was 216.

### Outcomes

This online survey tried to evaluate:

1. Dentists’ fear of becoming infected with COVID-19 practicing during the current COVID-19 pandemic.
2. Dentist’s knowledge about guidelines to fight the virus.
3. Various modifications in dental practice.

### Data sources and measurement

Data were gathered using an English language self-administrated questionnaire, which developed based on a similar study by Ahmed et al.^10^ after taking their permission. On the first page of the questionnaire, the participants were briefed about the purpose of the study and the voluntary and confidential nature of their participation. Informed consent was obtained from each participant in the form of answering a question (yes/ no) before proceeding with answering the questionnaire.

The questionnaire design consisted of 23 close-ended, multiple‐choice questions (yes/no/unaware or yes/no/don’t know) in three different sections. The first section of the questionnaire with four questions was related to the demographic data of the participants. The second section with 7 questions focused on the dentists’ fear of becoming infected with COVID-19 and the third section with 12 questions was designed to gather information regarding their knowledge about guidelines to fight the virus and to assess various modifications in dental practice.

### Bias

To limit selection bias, all dental professionals who participated in this study were randomly selected and invited to respond to a self-administered anonymous questionnaire.

To limit information bias, all participants were offered the same explanation regarding the nature and the aim of the study.

### Statistical methods

Collected data were analyzed by IBM-SPSS (Version 22.0) software package for Microsoft windows. Descriptive statistics were calculated as frequencies and percentages.

## Results

A total of 216 dental professionals completed the questionnaire. Most of them were general dental practitioners 113 (52.3%), below the age of 40 years with no sex predilection. Approximately about 146 (67.6%) of dentists enrolled were working in private clinics as illustrated in Table 1.

**Table 1:** Demographic characteristics of dental personnel in the study.

Characteristics	Number	Percentage
Gender		
Female	95	44.0%
Male	121	56.0%
Age		
20–30 years	88	40.7%
31–40 years	94	43.5%
41–50 years	15	6.9%
Above 50 years	19	8.8%
Designation		
General	113	52.3%
Specialist	86	39.8%
Consultant	17	7.9%
Working setting		
Private clinic	146	67.6%
Academic work or university	57	26.4%
Teaching or governmental hospital	93	43.1%

Fear of dental personnel during the COVID-19 outbreak was assessed using the questionnaire as shown in Table 2. A total of 200 (92.6%) dental professionals were afraid of becoming infected with COVID-19 while 196 (90.7%) became anxious to treat patients showing suspicious symptoms. About 151 (69.9%) wanted to close their practice until the number of COVID-19 cases starts declining and 156 (72.2%) felt nervous when talking to patients in close vicinity. Almost all respondents 213 (98.6%) were afraid of carrying the infection from dental practice to their families. Only 151 (69.9%) felt afraid of getting quarantined while 154 (71.3%) got anxious when heard about COVID-19 mortalities.

**Table 2:** Evaluation of fear of infection among dental personnel in the study.

Question	Number	Percent
Are you afraid of getting infected with COVID-19 from a patient and co-worker?		
Yes	200	92.6%
No	12	5.6%
Unaware	4	1.9%
Are you anxious when providing treatment to a patient who is coughing or showing suspicious symptoms?		
Yes	196	90.7%
No	18	8.3%
Unaware	2	0.9%
Do you want to close your dental practice until the number of COVID-19 cases starts declining?		
Yes	151	69.9%
No	58	26.9%
Unaware	7	3.2%
Do you feel nervous when talking to patients in close vicinity?		
Yes	156	72.2%
No	54	25.0%
Unaware	6	2.8%
Do you have a fear that you could carry the infection from your dental practice back to your family?		
Yes	213	98.6%
No	3	1.4%
Unaware	0	Zero
Are you afraid of getting quarantined if get infected?		
Yes	151	69.9%
No	62	28.7%
Unaware	3	1.4%
Do you feel afraid when you hear that people are dying because of COVID-19?		
Yes	154	71.3%
No	57	26.4%
Unaware	5	2.3%

Information regarding the knowledge of dental personnel about guidelines to fight the virus and various modifications in dental practice were also obtained using the questionnaire as illustrated in Table 3. Most of the respondents 203 (94.0%) were aware of the mode of transmission of COVID-19 but only 156 (72.2%) were updated with the current CDC or WHO guidelines for cross-infection control. Unfortunately, only 55 (25.5%) of dental personnel took the patient’s body temperature, 103 (47.7%) requested a patient’s travel history before performing any dental treatment, and only 154 (71.3%) deferred dental treatment of patients showing suspicious symptoms.

**Table 3:** The dentist’s knowledge about guidelines and various modifications in dental practice.

Question	Number	Percent
Are you aware of the mode of transmission of COVID-19?		
Yes	203	94.0%
No	6	2.8%
Don’t know	7	3.2%
Are you updated with the current CDC or WHO guidelines for cross-infection control regarding COVID-19?		
Yes	156	72.2%
No	38	17.6%
Don’t know	22	10.2%
Are you currently asking every patient’s travel history before performing dental treatment?		
Yes	103	47.7%
No	96	44.4%
Don’t know	17	7.9%
Are you currently taking every patient’s body temperature before performing dental treatment?		
Yes	55	25.5%
No	144	66.7%
Don’t know	17	7.9%
Are you deferring dental treatment of patients showing suspicious symptoms?		
Yes	154	71.3%
No	37	17.1%
Don’t know	25	11.6%
Do you think surgical mask is enough to prevent cross-infection of COVID-19?		
Yes	22	10.2%
No	180	83.3%
Don’t know	14	6.5%
Do you think N-90 mask should be routinely worn in dental practice due to the current outbreak?		
Yes	175	81.0%
No	24	11.1%
Don’t know	17	7.9%
Do you routinely follow universal precautions of infection control for every patient?		
Yes	183	84.7%
No	25	11.6%
Don’t know	8	3.7%
Do you use rubber dam isolation for every patient?		
Yes	53	24.5%
No	154	71.3%
Don’t know	9	4.2%
Do you use high-volume suction in your practice for every patient?		
Yes	104	48.1%
No	107	49.5%
Don’t know	5	2.3%
Do you wash your hands with soap and water and clean it using alcohol-based sanitizers before and after treatment of every patient?		
Yes	199	92.1%
No	14	6.5%
Don’t know	3	1.4%
Are you aware of which authority to contact if you come across a patient with suspected COVID-19 infection?		
Yes	134	62.0%
No	57	26.4%
Don’t know	25	11.6%

Regarding the use of personal protection, 180 (83.3%) of dentists believed that the surgical mask wasn’t enough to prevent cross-infection of COVID-19, and 175 (81.0%) believed that N-95 mask should be routinely worn. Although the majority of dentists 183 (84.7%) followed the universal precautions of infection control routinely, only 53 (24.5%) of the respondents reported the use of rubber dam isolation and 104 (48.1%) reported the use of high-volume suction. One hundred and ninety-nine (92.1%) of participants practiced washing hands with soap and water or sanitizer before and after treatment of patients, while 134 (62.0%) of participants were aware of the proper authority to contact if they came across a patient with a suspected COVID-19 infection.

## Discussion

This cross-sectional study assessed the fear of infection between Egyptian dentists practicing during the present COVID-19 pandemic and to explore their knowledge about guidelines to fight the virus and various modifications in dental practice through an online survey.

Data were gathered using a questionnaire based on the previous study of Ahmed et al.^10^ that was validated through intra-class correlation with a strong relation of 0.74. The online survey method is considered a useful tool for collecting data quickly with many benefits including saving time and money, ease of use, the capability to prohibit errors when entering and editing data, and rapid transmission of survey results.^12^

In a pandemic, fear, anxiety, and stress levels increase. Correspondingly, the rate of distress among healthcare staff is higher compared with the general population, because they are more at risk for infection.^9,13^

This is a result of the unique attributes of dental procedures including the proximity to the patients, the enormous number of droplets and aerosols generated during dental procedures,^1^ as the main route for transmission of coronavirus is through droplets and aerosols, the likelihood of dental professionals becoming infected and further spreading the virus is elevated.^10^

As a considerable viral load was consistently detected in the saliva of patients diagnosed with the infection by up to 91.7%,^1^ a high percentage of dentists reported their fear of becoming infected with COVID-19 from a patient or a co-worker in this study.

As usual protective measures in daily clinical practice are not effective enough to counter the spread of COVID-19, especially when patients are in the incubation period and are unaware that they are infected or prefer to conceal their infection,^7^ the majority of dentists stated being anxious when providing treatment to a patient who is coughing or showing suspicious symptoms. These findings triggered a large number of participated dentists to close their dental practice until the number of COVID-19 cases starts declining.

While dental professionals often accept the elevated risk of infection, as part of their chosen profession, they usually show concern about transmitting the infection to their families, especially if family members are elderly, immunocompromised, or possess a chronic medical condition. Almost all participants in the study feared for their families as they battle COVID-19, which can be linked to the fact that about 3000 healthcare providers in China became infected and transmitted the infection to family members. Despite the admission that transmission occurs mostly via symptomatic individuals, there are reports of asymptomatic individuals who transmitted the disease to multiple family members.^14,15^

These results were in accordance with the finding of Ahmed et al.,^10^ who reported that a large number of dentists fear becoming infected by their patients or co-workers and are also fearful of providing treatment to any patient reporting suspicious symptoms.

The majority of the respondents were aware of the mode of transmission of COVID-19 and many of them were updated with the current CDC or WHO guidelines for cross-infection control. However, a great number of participants reported not using rubber dam isolation or high-volume suction for every patient in their practice. Concerning the mode of transmission of COVID-19, the utilization of rubber dams can dramatically diminish the production of saliva- and blood-contaminated aerosol or spatter, especially when dental ultrasonic devices and high-speed handpieces are used. Also, it could significantly decrease airborne particles in the ~3-foot diameter of the operational area by 70%. The use of high-volume suction is also considered a crucial means to limit aerosols evacuation during dental treatment and should be used for most of the patients. Considering the benefits, there is no excuse for not using a rubber dam during dental procedures, especially while using rotary instruments.^16,17^

Many of the participants considered that the surgical mask wasn’t enough to counter the cross-infection of COVID-19 and that the N-95 mask should be routinely worn in dental practice during the current outbreak. It can be explained as surgical masks protect the oral and the nasal mucous membranes from droplet spatter, but they do not provide complete protection against the inhalation of airborne infectious agents unlike N-95 masks.^5,16^

Hand hygiene was performed by most of the dentists. Current evidence indicates that the COVID-19 virus is transmitted through respiratory droplets or contact. Contact transmission appeared directly when a contaminated hand contacted the mouth, the nose, or the eye; the virus can also be transmitted indirectly from one surface to another by contaminated hands. Therefore, hand hygiene is very important to prevent the spread of the COVID-19 virus.^18,19^

Conversations with front-line caregivers may help reduce anxiety. The focus should be on supportive communication, clear guidance when recommendations exist attempts to minimize misinformation, and efforts to reduce anxiety. Transparent and thoughtful communication could contribute to trust and a sense of control. Ensuring that workers feel they get adequate rest, can tend to critical personal needs and are supported both as healthcare professionals and as individuals will help maintain individual and team performance over the long run.^14,18^

## Limitation of the study

This research was subjected to some limitations:

- Time constraints: the rapid and extensive nature of the new coronavirus may intensify the respondent’s responses and feelings. Also, a change in their behavior might occur.
- Internet access: this study was conducted online so it was limited to participants who had internet access.

## Conclusions

COVID-19 pandemic has a significant impact on dental professionals. This is reflected in the high percentage of dental professionals experiencing fear and anxiety of getting infected while providing dental care. Some dental professionals often accepted the elevated risk of infection as part of their chosen profession and introduced several modifications in their dental practices in compliance with the recommended guidelines. While others want to close down their dental practice for a period of time until the number of COVID-19 cases starts declining. The majority of dental professionals were knowledgeable about the mode of transmission of COVID-19 and many of them were updated with the current CDC or WHO guidelines for cross-infection control.
